# Challenges in the Investigation of Therapeutic Equivalence of Locally Applied/Locally Acting Drugs in the Gastrointestinal Tract: The Rifaximin Case

**DOI:** 10.3390/pharmaceutics17070839

**Published:** 2025-06-27

**Authors:** Georgia Tsakiridou, Antigoni Maria Papanastasiou, Panagiotis Efentakis, Maria Faidra Galini Angelerou, Lida Kalantzi

**Affiliations:** Pharmathen SA, 31 Spartis Str., 14452 Metamorfosi Attica, Greece; gtsakiridou@pharmathen.com (G.T.); apapanastasiou@pharmathen.com (A.M.P.); pefentakis@pharmathen.com (P.E.); maggelerou@pharmathen.com (M.F.G.A.)

**Keywords:** locally applied and locally acting products, gastrointestinal track, bioequivalence, rifaximin

## Abstract

**Background:** Locally acting gastrointestinal (GI) drugs present challenges for generic drug development because traditional bioequivalence measures, which rely on systemic drug levels, do not reflect local efficacy. This review examines regulatory guidelines for establishing therapeutic equivalence for such drugs, using rifaximin—a minimally absorbed, gut-localized antibiotic—as a case study. **Methods:** We reviewed bioequivalence guidelines from the United States Food and Drug Administration (FDA) and European Medicines Agency (EMA), along with the literature on rifaximin’s biopharmaceutical and clinical properties, to identify strategies and challenges for establishing equivalence for locally acting GI drugs. **Results:** Rifaximin exemplifies the limitations of standard bioequivalence methods: as a Biopharmaceutics Classification System (BCS) class IV drug with minimal absorption and low solubility, in vitro dissolution may not predict local drug availability. Clinical endpoint trials (e.g., traveler’s diarrhea, hepatic encephalopathy, IBS-D) are resource-intensive and insensitive to formulation differences. Pharmacokinetic (PK) studies in healthy volunteers show low, variable plasma levels, which may inaccurately discriminate between formulations. The EMA requires evidence of non-saturable absorption to accept PK data, a difficult-to-establish but potentially irrelevant criterion. Differences between FDA and EMA approaches highlight a lack of harmonization, complicating global generic development. **Conclusions:** A tailored, multifaceted approach is needed to demonstrate bioequivalence for GI-localized drugs like rifaximin. This case underscores the need for more sensitive surrogate methods (e.g. advanced in vitro or pharmacodynamic models) and flexible regulatory criteria. Harmonization across international guidelines and innovative bioequivalence study designs are key to facilitating the approval of safe and effective generic alternatives in this drug class.

## 1. Introduction

Biopharmaceutically equivalent medicinal products are designed to mimic the performance and therapeutic effect of innovator drugs, through sameness in active ingredients, dosage form, and route of administration [1]. Two products are generally considered bioequivalent when they deliver the active pharmaceutical ingredient (API) to its site of action at a comparable rate and extent, without any clinically meaningful differences, when administered at the same molar dose under similar conditions [1,2]. The two major regulatory authorities, the U.S. Food and Drug Administration (FDA) and the European Medicines Agency (EMA), have outlined several approaches to demonstrate bioequivalence (BE), including therapeutic equivalence studies with clinical endpoints, in vivo BE studies with pharmacokinetic (PK) endpoints, in vivo BE studies with pharmacodynamic (PD) endpoints, and in vitro testing [1,2]. For drugs that are intended to enter systemic circulation, plasma/blood concentrations are readily measurable and have routinely served as surrogates for concentrations at the pharmacological site of action [3]. Accordingly, the demonstration of BE is straightforward, i.e., BE with the reference listed drug (RLD) is declared when the geometric mean ratio (GMR) and associated 90% confidence intervals (CIs) of PK metrics, such as the maximum concentration (C_max_) and the area under the curve from zero to the last sampling point (AUC_0–t_), fall within predefined limits (e.g., 80–125%) [2,4]. On the other hand, the demonstration of BE for locally applied and locally acting medicinal products intended for cutaneous, auricular, ocular, nasal, vaginal, and rectal use, or products acting locally within the gastrointestinal tract (GIT), presents a significant challenge, given that concentrations at the pharmacological site of action, in most cases, cannot be measured and plasma concentrations are not meaningful surrogates of local tissue concentrations. This issue becomes more pronounced for drug products acting locally in the GIT, and especially those acting in the intestine, as they may show no, limited, or complete absorption in the bloodstream, while the absorption site may or may not be the pharmacological action site [5,6]. The variable disposition kinetics and divergent mechanisms of action within this class of drug products, mean that a common methodological approach to demonstrate BE cannot be applied. The most common approach in the past was to prove therapeutic equivalence through clinical endpoint studies [7]. Still, BE studies with clinical endpoints have been heavily criticized as *“the least accurate, sensitive and reproducible of the general approaches for demonstrating bioequivalence”* [8], while at the same time being costly and time consuming [9]. The FDA has even provided a list of in vivo and in vitro methods to establish BE in descending order of preference: (1) in vivo studies in humans comparing drug/metabolite concentrations in an accessible biological fluid, (2) in vivo testing in humans of an acute PD effect, (3) controlled clinical trials in humans to establish safety and efficacy, (4) in vitro methods, and (5) any other approach deemed adequate by the FDA [8]. It could be inferred that BE studies with clinical endpoints are considered a less preferable option. While therapeutic equivalence studies with clinical endpoints have been the gold standard approach to show BE when PK studies are not appropriate, the aforementioned limitations encountered in therapeutic equivalence studies have shifted the way of thinking about these drug products within regulatory authorities (Ras) worldwide. As a result, a series of alternative approaches have recently been proposed to be more sensitive in detecting formulation differences and at the same time more manageable in terms of generic drug development timelines [10]. These alternative approaches are outlined within regulatory guidelines and Product-Specific Guidances (PSGs) developed by RAs, such as the FDA and EMA, considering the specific pharmaceutical parameters of each drug product, physicochemical characteristics of the API, and physiological processes at the site of action [5,6]. Within this paper, special focus is given to challenges associated with the clinical development of bioequivalent drug products intended to act locally in the GIT, taking into consideration RA requirements, and in particular, a detailed discussion on rifaximin is provided as a case example.

## 2. Regulatory Considerations on Products Acting Locally in the Gastrointestinal Track (GIT)

### 2.1. FDA Considerations on Products Acting Locally in the GIT

The FDA consistently publishes PSGs describing the agency’s current thinking and expectations on how to develop therapeutically equivalent products to specific reference listed drugs (RLDs). Seven case examples of FDA-recommended approaches for demonstrating the BE of locally acting GIT drugs as described in PSGs, along with drug product characteristics that have led to issuance of these recommendations, are presented within Table 1. These case examples belong to different types of products, such as binding agents, immediate (IR) and modified release products, products exhibiting varying degrees of systemic absorption and certainty on the in vitro prediction of in vivo behavior, as well as APIs with varying solubility and permeability parameters. It is evident that BE recommendations were generated in a drug-specific manner and were based on the ability, sensitivity, and reproducibility of the methodology to compare the rate and extent of active ingredient delivery at the site of action [11]. For example, binding agents Sevelamer Carbonate and Lanthanum Carbonate, which bind to dietary phosphate in the gut lumen and prevent its absorption [12], show minimal systemic absorption. Consequently, the binding agents’ concentration in circulation cannot be quantified accurately and is not indicative of its target engagement. On the contrary, the phosphorous binding equilibrium and kinetics can be quantitatively measured with validated in vitro methodologies. Hence, even though an in vivo study with clinical or PD endpoints could be in principle feasible, the in vitro option is proposed as a more accurate, efficient, and ethical alternative [13]. On the other hand, Orlistat, which acts as a gastrointestinal lipase inhibitor in the gut and is indicated for obesity management [14], shows minimal systemic absorption and exhibits low solubility, meaning that in vitro dissolution may not necessarily be relevant. Hence, the FDA proposes the use of the percent of fecal fat excretion as a PD endpoint to assess the BE of different formulations in comparison to the originator product [15], due to the accessibility and robustness of the measurement.

**Table 1:** FDA-proposed bioequivalence approaches and factors considered for seven cases of products acting locally in the intestine.

Product	Product Category	Indications	Strategy for Bioequivalence Investigation FDA	Systemic Absorption
***Sevelamer Carbonate***	Binding agent, binding can be quantitatively measured, local adverse events	For the control of serum phosphorus in adults and children 6 years of age and older with chronic kidney disease on dialysis [16]	Active ingredient sameness investigation and comparative in vitro binding studies [17]	No measurable concentrations in blood
***Lanthanum Carbonate***		Treatment of hyperphosphatemia in patients with end stage renal disease [18]	**Option 1:** Comparative in vitro dissolution and binding studies **Option 2:** In vivo bioequivalence study with PD endpoints [13]	0.002%
***Vancomycin***	Highly soluble active ingredient, product dissolution predictive of in vivo *(immediate release)*	Treatment of *Clostridium difficile*-associated diarrhea and enterocolitis caused by *Staphylococcus aureus* (including methicillin-resistant strains) [19]	**Option 1:** If the test product is qualitatively (Q1) and quantitatively (Q2) the same as the originator product: comparative in vitro dissolution **Option 2:** If the test product is not qualitatively (Q1) and quantitatively (Q2) the same as the originator product: in vivo study with clinical endpoints in patients with *Clostridium difficile*-associated diarrhea (CDAD) [20]	No measurable concentrations in blood
***Orlistat***	Low solubility active ingredient, PD endpoint easily measurable, dissolution not predictive of in vivo *(immediate release)*	Obesity management [21]	In vivo bioequivalence study with PD endpoints [15]	<2%
***Rifaximin***	Low solubility and low permeability, dissolution not predictive of in vivo *(immediate release)*	Traveler’s diarrhea caused by noninvasive strains of Escherichia coli in adult and pediatric patients 12 years of age and older (200 mg) and reduction in risk of overt hepatic encephalopathy recurrence in adults and irritable bowel syndrome with diarrhea (IBS-D) in adults (550 mg) [22]	**Option 1:** If the test product is qualitatively (Q1) and quantitatively (Q2) the same as the originator product, two bioequivalence studies with PK endpoints (1 fasting and 1 fed) per strength and comparative in vitro dissolution **Option 2:** If the test product is not qualitatively (Q1) and quantitatively (Q2) the same as the originator product, two bioequivalence studies with PK endpoints (1 fasting and 1 fed) per strength, comparative in vitro dissolution, and a bioequivalence study with clinical endpoints in patients with traveler’s diarrhea and a bioequivalence study with clinical endpoints in patients with IBS-D [23]	0.4%
***Mesalamine***	Dissolution predictive of in vivo *(modified release)*	Treatment of ulcerative colitis [24]	Two bioequivalence studies with PK endpoints (1 fasting and 1 fed) and in vitro comparative dissolution study [25]	20–30%
***Budesonide***		Treatment of ulcerative colitis [26]	Two in vivo bioequivalence studies with PK endpoints (1 fasting and 1 fed) and one in vitro comparative dissolution study [27]	10–20%

### 2.2. EMA Considerations on Products Acting Locally in the GIT

**Figure 1:**
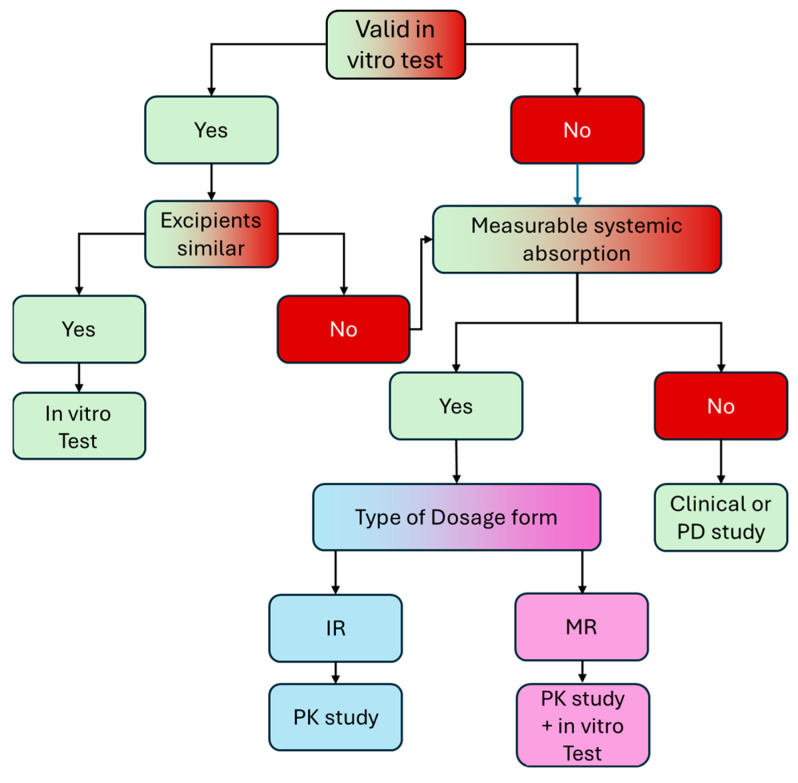
Decision tree on therapeutic equivalence approaches for solid dosage forms acting locally in the intestine, as proposed by EMA. Adapted from [31].

The EMA provided general guidance on the issue of BE investigation for products acting locally in the GIT within the 2019 guideline on equivalence studies for locally applied, locally acting products in the gastrointestinal tract [28]. In this guideline, it is recognized that *“alternative models (including in vitro and in vivo methods) may have a higher sensitivity than traditional clinical and pharmacodynamic (PD) endpoints studies to detect possible differences between medicinal products containing the same active substances”*. A stepwise approach is generally proposed by the EMA in the context of this guideline, allowing for several regulatory pathways to be followed, depending on the biopharmaceutical and PK properties of each product, while at the same time requiring extensive scientific rationale and justification. The decision tree proposed for the selection of the appropriate methodology for BE estimation for solid dosage forms acting in the intestine is shown in Figure 1.

As an example of harmonization between RA-recommended BE approaches, the EMA has also issued a PSG for Budesonide prolonged release products [29,30]; as per the PSG, similar to the FDA, two PK studies are proposed, one under fasting and one under fed conditions, while also including a range of partial AUCs as primary PK endpoints, in order to capture the drug product behavior at the clinically relevant absorption sites. On the other hand, an example of the absence of harmonization between RA-recommended approaches is the case of rifaximin, an antibiotic administered per os as an IR tablet and acting locally in the GI lumen. Rifaximin is a BCS IV compound with low solubility, low permeability, and a complicated disposition, with a fraction being present locally in the gut lumen and a fraction being absorbed in systemic circulation. In this review, through the use of rifaximin case study, a wide range of challenges affecting similar products can be identified.

## 3. Rifaximin Case Study

**Figure 2:**
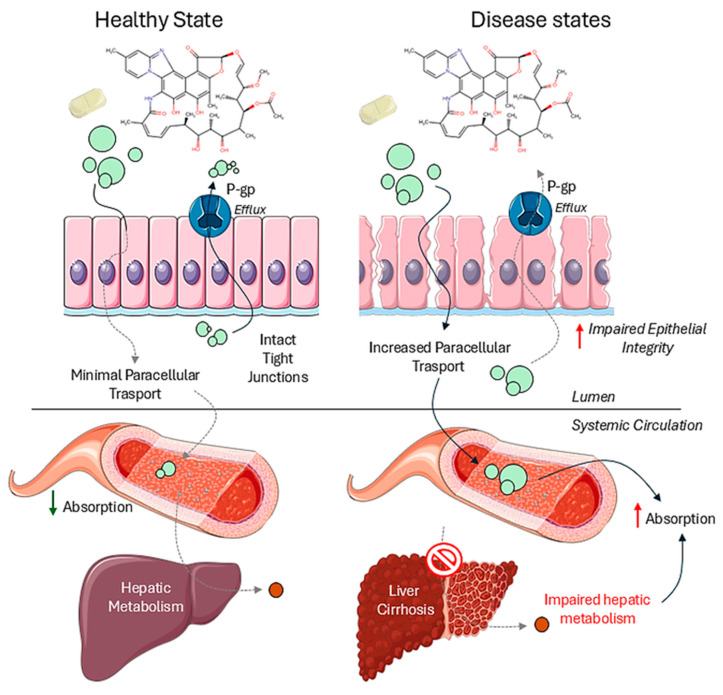
Rifaximin absorption in healthy (left panel) and disease states (right panel). Images were generated using templates from Servier Medical Art, licensed under a Creative Commons Attribution 3.0 Unported License. Chemical structures were created using Chemical Sketch Tool (Chemaxon^®^ v25.1.0, Protein Database). Red arrows represent an increase and green arrows represent a decrease in physiological parameters.

Rifaximin (C_43_H_51_N_3_O_11_, MW: 785.891 g/mol, Figure 2) is a semisynthetic derivative of rifamycin with antimicrobial properties [32], obtained by the reaction between rifamycin O and 2-amino-4-methylpyridine. Rifamycin O is a fermentation product from the Gram-positive filamentous actinomycete *Amycolatopsis mediterranei* [33]. Rifaximin has five known polymorphs, α, β, γ, δ, and ε [34], which can differ in key parameters, such as solubility, dissolution, and stability [35]. More specifically, rifaximin’s polymorphism demonstrates a wide range of aqueous solubilities. For instance, the β form exhibits the lowest solubility (~3.47 μg/mL), whereas the amorphous and mixed α forms show a higher solubility (ranging from 5.47 to 8.35 μg/mL), which is reflected in the drug’s bioavailability [36]. The α form, while used in commercial tablets, is known to convert into the more stable β form under humid conditions (>36% RH), which reduces dissolution efficiency over time [35]. Manufacturing conditions and excipients further influence these transformations—direct compression favors α form retention, while wet granulation can yield γ or other hydrated forms of rifaximin [37].

### 3.1. Summary of Rifaximin Commercial Products

Rifaximin was initially approved in Italy in 1987 for the treatment of a variety of gastrointestinal diseases, particularly diarrhea and portal systemic encephalopathy [38]. Since then, rifaximin has received approval in several EU countries through the decentralized process, under different brand names and marketing authorization holders, i.e., XIFAXANTA^®^, XIFAXAN^®^ (Norgine B.V., Amsterdam, Netherlands), Refero^®^, NORMIX^®^, Tixteller^®^ (AlfaSigma S.p.a., Bolognia, IT, US), and others [39]. In 2005, it received approval from the FDA for the treatment of travelers’ diarrhea, secondary to non-invasive *Escherichia coli*, under the name XIFAXAN^®^ (Salix Pharmaceuticals, New Jersey, USA). In 2010 and 2012, rifaximin received, both in the US and in several EU countries, respectively, a new labeling for the reduction in the risk of recurrence of overt hepatic encephalopathy in patients with advanced liver disease aged 18 years or older [40]. Finally, in 2015, rifaximin was approved in the US for the treatment of irritable bowel syndrome with diarrhea (IBS-D) in adults [41]. Rifaximin is available as film-coated tablets in two strengths, 200 mg and 550 mg. The 200 mg strength tablet is administered orally three times a day for 3 days for the treatment of traveler’s diarrhea [42], while the 550 mg strength tablet is administered twice daily for the treatment of hepatic encephalopathy [41,43] and three times daily for 14 days for the treatment of IBS-D [41]. Commercial product strengths, indications, and posology per territory are summarized in Table 2.

### 3.2. Rifaximin Pharmacokinetic and Pharmacodynamic Characteristics

Rifaximin is a BCS class IV, low-solubility, low-permeability compound [44,45,46], with low absorption following oral administration, and the GIT as the primary target organ. Its retention in the intestine is also facilitated by its hydrophobicity and ionization at all pH values along the GIT [47]. Rifaximin plasma concentrations are low and variable, with ~3% of the dose being absorbed in healthy volunteers but only 18% of that (i.e. ~0.4% of dose) reaching circulation due to metabolism in liver by the CYP3A4 enzyme and its interaction with the P-glycoprotein (P-gp)-mediated efflux transport mechanism [44,45,46]. After repeated dosing, peak plasma levels range consistently from 0.68 to 3.4 ng/mL [41]. Generally, plasma levels remain below 10 ng/mL in healthy individuals and patients with intestinal mucosa damage [42]. However, in hepatic encephalopathy patients, systemic exposure is about 12 times higher than in healthy subjects on the same dose, possibly attributed either to a compromised liver-dependent metabolism [41] or to the impaired integrity of the intestinal epithelial barrier in patients with hepatic encephalopathy (such as gastrointestinal bleeding) [48], possibly leading to a greater translocation of substances, including medications like rifaximin, into systemic circulation (Figure 2). A high-fat meal taken within 30 min of dosing causes a clinically insignificant increase in absorption [42]. Rifaximin shows high variability in pharmacokinetics. Inter-subject variability for AUC and C_max_ ranges from 37% to 89% [49,50], translating to an estimated intra-subject variability of 25% to 60%. The literature also supports an intra-subject variability of 30% or more for both AUC and C_max_ [51].

**Figure 3:**
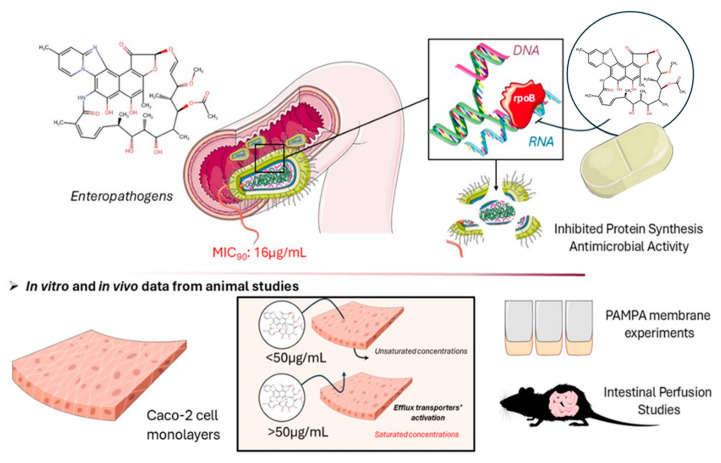
Chemical structure and mechanism of action of rifaximin and absorption. Images were generated using templates from Servier Medical Art, licensed under a Creative Commons Attribution 3.0 Unported License. Chemical structures were created using Chemical Sketch Tool (Chemaxon^®^, Protein Database).

Rifaximin exhibits a broad spectrum of in vitro and in vivo activity [52] and modulates microbial virulence [53,54] and epithelial cell function [55]. It has been shown to have both bactericidal and bacteriostatic properties, with minimal effects on the colonic bacterial flora [56] and without facilitating significant changes in gut microbiota composition [57]. Several in vitro studies have shown that rifaximin displays inhibitory activity against Gram-positive, Gram-negative, aerobic, and anaerobic bacteria [47,58]. The PD effects of rifaximin, in all three indications, are attributed to the reduction in bacteria populations, including gas-producing bacteria [59]. More specifically, rifaximin acts locally in the GIT prior to absorption in systemic circulation, by irreversibly binding to the bacterial DNA-dependent RNA polymerase called rpoB, thus inhibiting the bacteria’s ability to synthesize proteins, resulting in the reduction in harmful bacteria populations within the intestine (Figure 3) [60,61].

As with other antimicrobials, rifaximin’s efficacy is linked to achieving minimum inhibitory concentrations in the site of action (MIC, expressed in mg/L (μg/mL)) [62,63]. More specifically, in vitro susceptibility studies demonstrated that the MIC_50_ and MIC_90_ values of rifaximin—representing the concentrations required to inhibit 50% and 90% of isolates, respectively—ranged from 4 to 8 μg/mL and 4 to 16 μg/mL for the majority of 177 bacterial enteropathogens linked to traveler’s diarrhea (Figure 3) [62]. However, for locally acting agents like rifaximin, MIC values have to refer to luminal rifaximin concentrations in the intestine [39,64]. That being said, though, due to the absence of direct intraluminal drug concentration data for rifaximin, MIC interpretation remains limited. Nonetheless, available data suggest that luminal concentrations far exceed MIC values; for instance, fecal levels after a three-day oral administration regimen range from 4000 to 8000 μg/g, approximately 160–250 times higher than the reported in vitro MIC_90_ for common enteropathogens [65,66].

### 3.3. Regulatory Framework for the Approval of Rifaximin Bioequivalent Products; The FDA and EMA Approaches

#### 3.3.1. FDA Approach to the Approval of Rifaximin Bioequivalent Products

The FDA’s approach to establishing BE for rifaximin has evolved significantly over time, moving from the first PSGs in 2011–2012, proposing the conduct of therapeutic equivalence studies with clinical endpoints, to a combination of PK and in vitro dissolution studies in 2022.

More specifically, the agency initially issued separate PSGs for the 200 mg and 550 mg strengths. The 200 mg product required a clinical endpoint study in patients with traveler’s diarrhea [67], while the 550 mg product could rely on the clinical efficacy data from the 200 mg strength, supplemented by a fasting and a fed PK study [68]. In 2017, the FDA consolidated this guidance into a single revised draft PSG [69], reflective of advances in bioanalytical methods. In case the product is qualitatively and quantitatively (Q1/Q2) similar to the originator formulation, the new guidance is allowed for the demonstration of BE for the 200 mg product through PK studies (fasting and fed) and in vitro dissolution testing. In the case that the 200 and 550 mg formulations showed dose-proportional formulation characteristics, a biowaiver for the PK study of the 550 mg product could be supported. However, if the products were not Q1/Q2 to the originator, a combination of PK, clinical, and in vitro studies remained necessary.

In 2022, based on new information becoming available, suggesting that the 200 and the 550 mg products do not exhibit dose proportionality, the FDA further updated the PSG [23] to eliminate the biowaiver option for the 550 mg product and to require fasting and fed PK studies along with dissolution testing for both strengths in Q1/Q2 equivalent formulations. For non-Q1/Q2 equivalent products, the FDA reinstated the requirement for clinical endpoint studies for both strengths. Despite these changes, the FDA reiterated its position that, based on the totality of evidence available, PK and in vitro dissolution methods remain more appropriate than clinical endpoint studies for demonstrating BE whenever scientifically justified.

#### 3.3.2. EMA Approach to the Approval of Rifaximin Bioequivalent Products

The EMA has not issued PSGs for rifaximin products. Accordingly, recommendations for the investigation of BE can be followed from the guideline on equivalence studies for locally applied, locally acting products in the GIT section 4.3.3 [28], which outlines the EMA’s approach for drugs acting in the intestine. Rifaximin’s site of action is the entire length of the lumen of the small intestine and thus, as a solid dosage form showing levels of systemic absorption, falls in principle in the category of drug products for which BE can be investigated through fast and fed PK studies. This approach is only applicable when “*absorption is not saturated (demonstrated* e.g., *by means of a dose-proportionality study)*”. Otherwise, in the absence of evidence that absorption is not saturated, BE should be investigated through appropriate studies with clinical endpoints.

## 4. Discussion

Both the EMA and FDA describe the possibility of supporting the clinical development program of a bioequivalent rifaximin product with a set of PK studies if certain strict criteria are met, such as non-saturable absorption for the EMA and Q1/Q2 similarity for the FDA. If these criteria are not met, a clinical endpoint study is required.

### 4.1. Bioequivalence in Clinical Endpoints

In the case of rifaximin, the logistics of a clinical endpoint study is very challenging due to the study populations eligible for participation: patients with traveler’s diarrhea for the 200 mg product (for both EU and US submission), patients with liver cirrhosis who have already experienced an episode of hepatic encephalopathy for the 550 mg product for EU submission, and patients with a clinical diagnosis of irritable bowel syndrome with diarrhea (IBS-D) for the 550 mg product for US submission. In the first case, a study in patients with traveler’s diarrhea would require a sample size of approximately 300–750 patients with an expected duration of 1–3 years (sometimes spread within two or three holiday seasons) [70,71]. As a result, some studies have been prematurely terminated due to recruitment issues [72]. In the second case, 400–600 hepatic encephalopathy patients would be needed for bioequivalence studies with clinical endpoints that can last up to 3–4 years [73,74]. On the other hand, even though equivalence studies in patients with IBS-D can be concluded with approximately 150–200 patients in 3–6 months [75], clinical investigation is not straightforward. More specifically, the study design along with a strong response to placebo have created obstacles for effective clinical trials in this population [76,77]. Consequently, the interpretation of study results can be not only challenging but also potentially biased. Hence, BE studies in the aforementioned patient populations are challenging design-wise, time- and cost-consuming, and sometimes not even feasible, creating potentially unsurpassable obstacles in the approval of generic products.

### 4.2. Bioequivalence in Pharmacokinetic Endpoints

The option of an in vivo study with PK endpoints is in principle a more realistic approach for rifaximin, even though simultaneously challenging and potentially over discriminating. On top of this, the prerequisites that are set by the agencies allow for the investigation of BE through studies with PK endpoints, i.e., non-saturable absorption for the EMA and Q1/Q2 similarity for the FDA, are of rather doubtful relevance.

More specifically, rifaximin exhibits an excellent safety profile due to its limited absorption into systemic circulation [78]. Hence, studies with PK endpoints can be conducted in healthy volunteers, rather than patients who are needed in the clinical endpoint studies. In addition, PK studies provide a more standardized cost- and time-effective approach for the investigation of BE, further supporting the argument that they are a more realistic option for the clinical program of rifaximin products. However, rifaximin exhibits very low bioavailability and high variability in terms of relevant PK metrics, features that add a challenging aspect to the potential use of PK studies for the investigation of BE. This is especially evident as the EMA [2], in contrast to the FDA [4], does not allow widening of the 90% CIs for the AUC metric when the intra-subject variability is more than 30%. However, due to the fact that rifaximin exerts its therapeutic effect by achieving intraluminal concentrations higher than its MIC_90_ values for relevant enteropathogens (~16 μg/mL) [62], differences in the intraluminal concentrations above the local MIC would lead to differences in PK metrics with no clinical relevance; thus, BE criteria would be met, even though two products are therapeutically equivalent. Thus, it could be proposed that hurdles imposed by the PK study methodology may potentially create an over-discrimination bias against bioequivalent products.

Additionally, even the prerequisite conditions that would allow for the PK clinical development option to be followed, as per the EMA and FDA, are arbitrary. More specifically, regarding the condition that absorption should be non-saturable, as imposed by the EMA, extensive literature data on the topic are lacking, as due to the local action of rifaximin, absorption has not been investigated in formal proportionality studies in humans as part of the submission dossier of the originator product. To the best of our knowledge, all publicly available human data on the investigation of the dose-proportionality of rifaximin products are depicted in Table 3. Available data are limited and scrutinized by the high variability in the PK metrics, making the extrapolation of conclusions difficult. At the same time, any attempts to explore the range of non-saturable absorption in animal models is compromised by the difficulty to identify the most representative animal model for this task, due to rifaximin’s physicochemical characteristics of low solubility and low permeability, which create a unique interplay [79], and the lack of consensus between scientists on the best surrogate human PK animal model for this type of molecule.

Healthy volunteers were dosed with 200 mg (in the form of a single tablet of the 200 mg product from US market), 400 mg (in the form of two tablets of the 200 mg product), 600 mg (in the form of three tablets of the 200 mg products), or 550 mg (in the form of a single tablet of the 550 mg product form US market) of rifaximin, using the originator formulations. The mean dose-AUC until the last sampling point (AUC_0–last_) for the 200, 400, and 600 mg doses are calculated to be dose-proportional, as the difference in dose-adjusted mean AUC is less than 25% when comparing the range of strengths proposed (ICH M13A). Dose-proportionality is not true between the 200 and 550 mg product from US market, as the difference in dose-adjusted mean AUC for the two products exceeds the 25% threshold. Data are presented as mean ± SD.

### 4.3. Site-of-Action Considerations in Rifaximin Bioequivalence

Generally, it is accepted that rifaximin’s permeability is low and there is evidence that it is limited by rifaximin’s concentration in the intestinal lumen as well as transport mechanisms (i.e. P-gp efflux and/or BCRP, MRP2 and/or basolateral uptake transporters) [44,50]. Importantly, data derived from in vitro and in vivo animal models indicate that the maximal concentration of rifaximin that is linearly absorbed is 50μM (~53 μg/mL) (above this concentration, interaction with efflux transporters is initiated) [44,50], which is three times higher than the effective MIC_90_ values against enteropathogens (~16 μg/mL, [62]). Therefore, it can be hypothesized that rifaximin retains the same clinical efficacy through a range of saturable and non-saturable concentrations, making the EMA’s prerequisite for non-saturable absorption as a decision-making clinical strategy tool irrelevant for products whose therapeutic effect relies on surpassing a concentration value which is robustly lower than the linearity limit.

On the other hand, regarding the prerequisite for products to be Q1/Q2 in order to follow the PK route, as per the FDA PSG, it is evident that the condition aims to ensure comparable dissolution and solubility behavior in the gut. Generally, potentially different functional excipients could lead to alternate/different equilibrium concentrations in the gut. However, given that the rifaximin concentration in feces after multiple oral administrations reaches levels 160–250 times higher than the reported therapeutic effect threshold of in vitro MIC_90_ [65,66], it is highly unlikely that certain formulation differences could lead to meaningful therapeutic effect differences.

Finally, recognizing the need for scientific reassurance for safe and effective medicines, it becomes clear that deciphering in vivo concentrations at the site of action is of outmost importance, not only for the investigation of the BE of rifaximin products but also for the deeper understanding of the behavior of locally acting products in the intestine. Even though techniques imaging the dissolution of the products in the GIT (i.e. pharmacosintigraphy) have been employed previously to supplement the investigation of the BE of other locally acting products in the intestine [81,82,83,84], they cannot provide adequate information on the concentrations of active ingredients at each site and hence cannot be considered standalone approaches [85]. This is due to the primarily qualitative data generated by pharmacosintigraphy, which provide information on the transit, disintegration, and release patterns of orally administered radiolabeled formulations, whilst the actual concentration of the active pharmaceutical ingredient (API) at specific sites within the GIT is not quantified [85].

Importantly, directly measuring concentrations in the gut lumen can be challenging to perform on a wide scale for the investigation of BE [86], but it might be useful as a validation tool for in vitro dissolution methodologies for the prediction of intraluminal rifaximin concentrations. Gut content aspiration studies have been conducted previously for another locally acting agent used for the treatment of ulcerative colitis, mesalamine [87,88]. The availability of these data resulted in the updated mesalamine PSG, where appropriate in vitro dissolution testing conditions were recommended for each section of the intestine, along with two PK studies [25]. This work also gave rise to the validation of physiologically based PK modeling (PBPK) applications, which aimed to predict the concentrations of new generic mesalamine formulations in sections of the intestine [89,90]. It is clear that for any pivotal activities involving in vitro methodologies, guidance from regulatory authorities is imperative.

### 4.4. Future Considerations

Based on the above, it is evident that rifaximin products present a unique challenge for BE assessment, with significant obstacles arising in both PK and clinical endpoint studies. This complexity is reflected in the fact that, despite the expiration of data exclusivity in the EU several years ago, no bioequivalent product/drugs have been brought to the market. Similarly, in the United States, although the FDA has granted tentative approval to several ANDA submissions, product launches have been deferred until 2029 due to patent litigation procedures—likely related to formulation-specific aspects such as Q1/Q2 equivalence.

Given these challenges, there is a clear need for alternative approaches that can more effectively capture both intraluminal drug concentrations and PD effects related to bacterial eradication. One such approach could involve the use of in vitro bacterial susceptibility models, which track changes in colony density over time following exposure to biorelevant concentrations of rifaximin. However, to date, such assays have primarily been limited to basic research settings, largely due to difficulties in achieving acceptable validation for regulatory submissions. Future work should focus on standardizing and validating these models to support their integration into regulatory frameworks for locally acting gastrointestinal drug products.

## 5. Conclusions

Demonstrating bioequivalence for acting locally in GIT drugs such as rifaximin requires a delicate, multifactorial strategy. This case study of rifaximin exemplifies the need to develop more sensitive surrogate methodologies—such as advanced in vitro systems or pharmacodynamic models—and adopt more adaptable regulatory frameworks. Progress in international guideline harmonization, along with innovative study designs, will be critical to supporting the approval of safe and effective generic alternatives within this therapeutic class.

## Figures and Tables

**Table 2:** Summary of commercial product strengths, indications, and posology per territory.

Territory	Strength	Indication	Posology
US	200 mg	Treatment of travelers’ diarrhea (TD) caused by noninvasive strains of Escherichia coli in adult and pediatric patients 12 years of age and older	One 200 mg tablet 3 times a day for 3 days
	550 mg	Reduction in risk of overt hepatic encephalopathy (HE) recurrence in adults	One 550 mg tablet 2 times a day
		Treatment of irritable bowel syndrome with diarrhea (IBS-D) in adults	One 550 mg tablet 3 times a day for 14 days
EU	200 mg	Treatment of traveler’s diarrhea that is not associated with any of the following: fever, bloody diarrhea, eight or more unformed stools in the previous 24 h, occult blood or leucocytes in the stool	200 mg every 8 h for three days (total 9 doses)
	550 mg	For the reduction in recurrence of episodes of overt hepatic encephalopathy in patients ≥ 18 years of age	550 mg twice a day as long-term treatment for the reduction in recurrence of episodes of overt hepatic encephalopathy

**Table 3:** Dose proportionality assessment of rifaximin AUC. Data adapted from [80].

Rifaximin	AUC_0–last_ (h × ng/mL) Mean (SD)	Dose Normalized AUC	% Difference of Dose Corrected AUCs (AUC200 mg-AUCxmg)/AUC200 mg
200 mg	3.23 (1.55)	0.016	N/A
400 mg	6.23 (3.04)	0.016	0%
600 mg	7.76 (4.69)	0.013	18.75%
550 mg	3.65 (3.66)	0.007	56.25%

## Data Availability

No new data were created or analyzed in this study. Data sharing is not applicable to this article.

## Abbreviations

The following abbreviations are used in this manuscript:

API	Active Pharmaceutical Ingredient
AUC	Area Under the Curve
AUC_0_–t	Area Under the Curve from zero to the last sampling point
AUC_0_–last	Area Under the Curve until the last sampling point
BCS	Biopharmaceutics Classification System
BE	Bioequivalence
BCRP	Breast Cancer Resistance Protein
CDAD	Clostridium difficile-Associated Diarrhea
CI	Confidence Interval
Cmax	Maximum Concentration
EMA	European Medicines Agency
FDA	Food and Drug Administration
GI	Gastrointestinal
GIT	Gastrointestinal Tract
GMR	Geometric Mean Ratio
HE	Hepatic Encephalopathy
IBS-D	Irritable Bowel Syndrome with Diarrhea
IR	Immediate Release
MIC	Minimum Inhibitory Concentration
MIC_50_	Minimum Inhibitory Concentration to inhibit 50% of isolates
MIC_90_	Minimum Inhibitory Concentration to inhibit 90% of isolates
MW	Molecular Weight
PD	Pharmacodynamic
PK	Pharmacokinetic
PSG	Product Specific Guidance
P-gp	P-glycoprotein
Q1	Qualitative Sameness
Q2	Quantitative Sameness
RLD	Reference Listed Drug
RA	Regulatory Authority
rpoB	RNA Polymerase Beta Subunit
SD	Standard Deviation
TD	Traveler’s Diarrhea
